# Phase angle in localized bioimpedance measurements to assess and monitor muscle injury

**DOI:** 10.1007/s11154-023-09790-9

**Published:** 2023-02-27

**Authors:** Lexa Nescolarde, Antonio Talluri, Javier Yanguas, Henry Lukaski

**Affiliations:** 1 Department of Electronic Engineering, Universitat Politècnica de Catalunya, c/ Jordi Girona 1-3, Edifici C4, 08034 Barcelona, Spain; 2 PixelCanDo, Santa Cruz, Tenerife Spain; 3 Futbol Club Barcelona, Ciutat Esportiva Joan Gamper, Av. c/ Onze de Setembre s/n, 08790 Sant Joan Despí, Barcelona, Spain; 4 Department of Kinesiology and Public Health Education, University of North Dakota, Grand Forks, ND 58202 USA

**Keywords:** Localized bioimpedance, Muscle, Reactance, Magnetic resonance imaging

## Abstract

Localized bioimpedance (L-BIA) measurements are an innovative method to non-invasively identify structural derangement of soft tissues, principally muscles, and fluid accumulation in response to traumatic injury. This review provides unique L-BIA data demonstrating significant relative differences between injured and contralateral non-injured regions of interest (ROI) associated with soft tissue injury. One key finding is the specific and sensitive role of reactance (Xc), measured at 50 kHz with a phase-sensitive BI instrument, to identify objective degrees of muscle injury, localized structural damage and fluid accretion, determined using magnetic resonance imaging. The predominant effect of Xc as an indicator of severity of muscle injury is highlighted in phase angle (PhA) measurements. Novel experimental models utilizing cooking-induced cell disruption, saline injection into meat specimens, and measurements of changing amounts of cells in a constant volume provide empirical evidence of the physiological correlates of series Xc as cells in water. Findings of strong associations of capacitance, computed from parallel Xc (X_CP_), with whole body counting of 40-potassium and resting metabolic rate support the hypothesis that parallel Xc is a biomarker of body cell mass. These observations provide a theoretical and practical basis for a significant role of Xc, and hence PhA, to identify objectively graded muscle injury and to reliably monitor progress of treatment and return of muscle function.

## Introduction

Whereas whole-body tetrapolar phase-sensitive bioimpedance measurements characterize various parameters of body composition, risk of malnutrition and prognosis, site-specific tetrapolar phase-sensitive bioimpedance (BI) measurements provide objective information describing the effects of severity of soft tissue traumatic injury and recovery unavailable from whole-body measurements. Nyboer et al. [1] first proposed localized bioimpedance (L-BIA), an innovative technique described as a “segmental configuration” in contrast to the whole-body electrode arrangement. They applied aluminium foil electrodes positioned with two current-injecting electrodes located on the outer aspect of the region of interest (ROI) and two voltage-detecting electrodes adjacent to the ROI and separated by 20 cm. This pioneering approach provided the foundation for future applications of L-BIA that utilize a 50 kHz phase-sensitive BI instrument and four contact electrodes positioned near the anatomical ROI to optimize determinations of resistance (R), reactance (Xc) and phase angle (PhA) that allow for assessment of anatomical structure and physiological status.

Contemporary applications of the L-BIA method focus on muscle injury. This review aims to describe a novel application of L-BIA to identify, classify and monitor injury in skeletal muscle. It addresses critical issues related to the technical needs of BI devices to measure R, Xc, and PhA *in vivo*, provides empirical evidence of physiological correlates for BI measurements, reviews practical applications for use of L-BIA to classify muscle injury and to discriminate tissue damage from localized fluid accumulation, and demonstrates the practical use of L-BIA to establish objective indexes for determination of healing for return to physical activity in athletes.

## Resistor–capacitor cell models: interpretation of bioimpedance measurements and optimal phase detection frequency

**Fig. 1:**
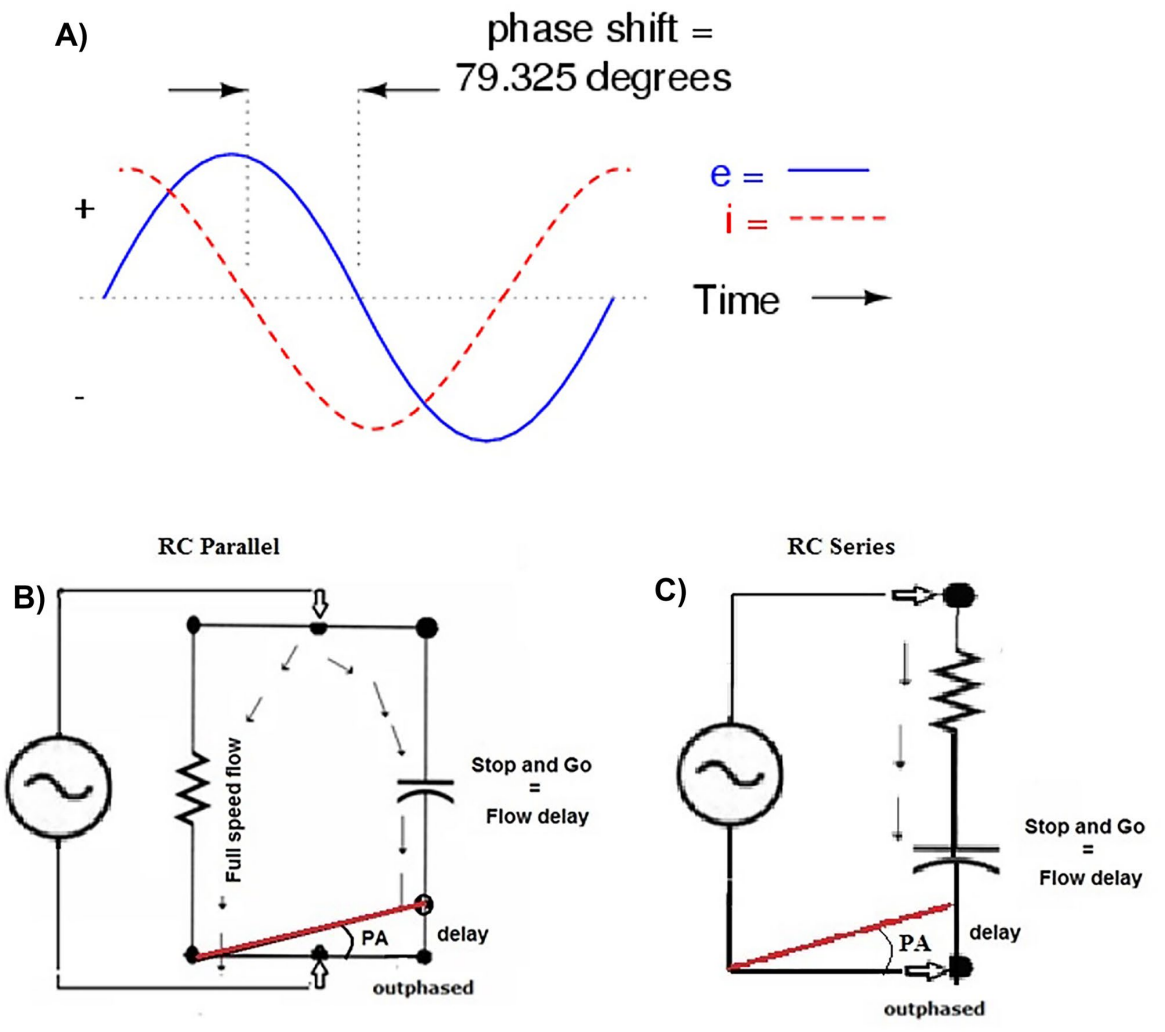
Phase-angle shift of sinusoidal current (i) and voltage (e) (**A**). Current flow velocity according to RC parallel cell model (**B**) and RC series cell model (**C**)

A basic understanding of the conduction of applied electrical current is required to interpret physiological significance of BI measurements. The human body may be considered as a network of resistors and capacitors represented as a resistor–capacitor (RC) equivalent circuit [2]. Introduction of an alternating electrical current (AC) divides into conductive (water and electrolytes) and capacitive (cell membranes and tissue interfaces) pathways. If a conductor is purely resistive, the flow of the current is continuous, without any time delay, thus the current and voltage are flowing in phase. Because the applied current is alternating (AC), any impairment of current flow is termed impedance, and it is appropriate to use the term resistive impedance (R). If within the circuit, non-conductive elements such as capacitors are present, current flow can be delayed resulting in a time delay or phase shift, which is the lag between current and voltage (Fig. 1A).

Measurement of BI parameters as surrogates of biological entities assumes the presence of a capacitor-equivalent component in the circuit consistent with "cellular mass" that has a structure behaving as a capacitor. This model includes a conductor enclosed by insulating structures and surrounded by another conductor. The corresponding physiological resistive components are the intracellular water (ICW) and extracellular water (ECW) with the insulating lipid-containing cell membrane, reactive as a capacitor.

The location of a capacitor determines the type of RC equivalent circuit. A parallel RC has the capacitor separate or aside a purely resistive conductor (Fig. 1B), and reflects the physiological model of cells in tissues. If the capacitor is in-line with a purely resistive conductor, the circuit is series equivalent (Fig. 1C) not reflecting correctly the physiological model.

Experiments to ascertain physiological equivalents of BI measurements utilize two methods. One protocol expands the extracellular space with addition of conductive fluids, decreasing the less resistive branch parallel to the capacitive side of the circuit. Another approach is to disrupt the cell membranes with temperature (e.g., heat or freezing), damaging or destroying the capacitive branch thus decreasing or eliminating the phase shift. Series and parallel cell models include Xc, which indicates the opposition to the AC flow due to cell membranes that act as capacitors (Fig. 1B, C). Regardless of the reference model (series or parallel), the overall Z is always the same. Hence, the value of Z does not change, only the values of the two components (R and Xc) are substantially different [2].

The value of Xc is frequency-dependent and is described as capacitive resistance, which is inversely related to frequency and CAP. All BI devices reported on the displays Xc and R series which necessitates being transformed in equivalent parallel as Rp and Xcp, to comply with tissue physiological model.

The transforms are the following:

$${\mathrm{R}}_{\mathrm{p}}={R}_{50kHz}+\frac{{{X}_{C}}_{50kHz}^{2}}{{R}_{50kHz}}$$

$${\mathrm{X}}_{\mathrm{Cp}}={X}_{C50kHz}+\frac{{R}_{50kHz}^{2}}{{X}_{C50kHz}}$$

The capacitance (CAP) generating the parallel (Xcp), when stimulated at 50 kHz of the parallel cells in the parallel Resistive (Rp) fluids, can be computed as:

$$CAP=\frac{1\mathrm{E}12}{{\mathrm{f}}_{50kHz}*{\mathrm{X}}_{\mathrm{Cp}}*2\pi }=\frac{1\mathrm{E}12}{50000*{\mathrm{X}}_{\mathrm{Cp}}*6.28}\;(^{*}1\mathrm{E}12:\mathrm{scientific\;notation})$$

The type of bioelectrical model affects the physiological interpretation of Xc. Studies of variable red blood cell concentrations (hematocrit; HCT) in a constant volume of saline reveal a linear relationship between HCT and CAP [3] at the same temperature. This observation indicates that series Xc, which is reported by commercial BI devices, is indirectly related to the concentration of cells in water. Parallel Xc (X_CP_), however, provides additional fluid and cell information and describes cells and ICF quantitatively [4]. Estimating the body cell mass (BCM) from BI parallel cell models used tetra-polar whole-body BI measurements, in healthy elders, at single-frequency (SFBIA: 50 kHz) and multifrequency Z measurements (MFBIA: 1, 5, 50, 100 kHz) with phase detection only at 50 kHz (BIA 2000-M, Data Input, Hofheim, Germany) and whole body ^40^ K counting as a reference method. The X_CP,_ was obtained from series Xc reported from STA-BIA (Akern Srl, Florence, Italy) [4]. Validation of parallel BI models found no differences of SF or MF estimates with reference BCM values. These findings enable a practical reasoning of series Xc as related to hydration and parallel Xc as an index of BCM.

More data are necessary to consolidate the power of the series to parallel transforms. However, PhA values are identical for series and parallel circuits. Thus CAP, as derived from a parallel circuit model, is appropriate for physiological equivalence.

**Fig. 2:**
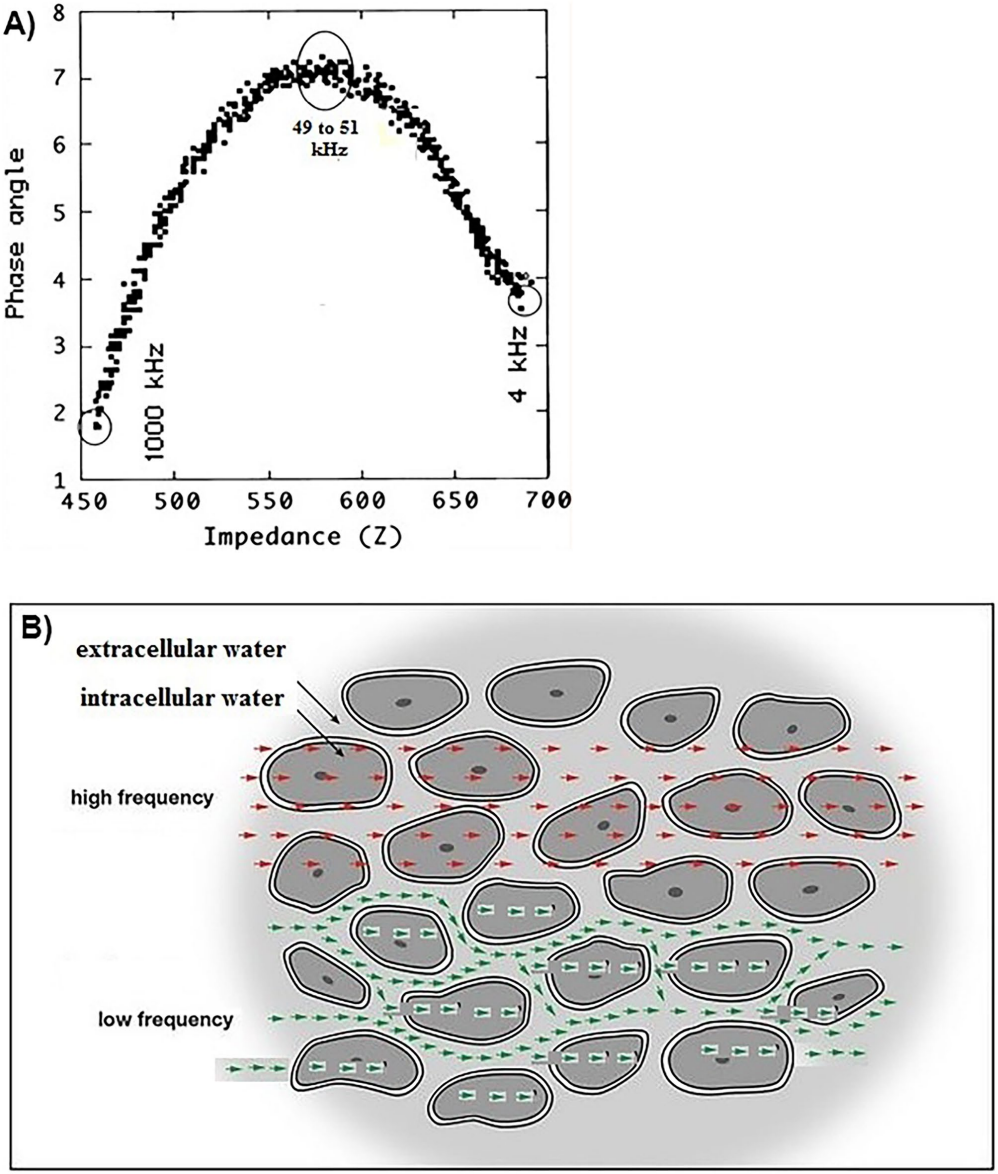
Effects of frequency on phase angle from 4 to 1000 kHz using a SFB3 multifrequency bioimpedance instrument (**A**). Alternating current circulation is within the biological tissue (**B**)

The literature often contains statements that, at low frequencies (~5 kHz), the electrical current flow occurs only in the ECW (resistive) pathway but at higher frequencies (> 5 kHz) the current penetrates (circulates) also inside the capacitive component (cells), thus corresponding to the whole-body water conductor (Fig. 2A) and causing a phase shift. However, the sole way to have only extracellular circulation is to apply direct current (DC). Applying an AC signal at any frequency will produce a permittivity (dielectric propriety of biological tissue) through any capacitor. Both permittivity and conductivity, are tissue-type and frequency-dependent and closely related to the cell membrane [5].

Experimental data [6] obtained with a SFB3 impedance spectroscopy device (SEAC-UniQuest, Australia) demonstrate that low frequency AC (4 kHz) penetrates the cell membrane and enables measurement of lower levels of Xc and PhA values compared to 50 kHz AC (Fig. 2A). Therefore, it is more appropriate to describe the effect of frequency on conduction of AC as shown in Fig. 2B.

The frequency at which the current is administered contributes to value of PhA measurements. In healthy adults, 50 kHz is the average frequency at which Xc is maximal (Fig. 2A). At this frequency, alternating current flows predominantly in the extracellular space but some can cross the cell membrane [7, 8]. Importantly, PhA decreases at lower and higher frequencies (Fig. 2A). Thus, PhA is frequency-dependent, due to the number of cells in parallel to the ECW (e.g., concentration of cells in water), which is only resistive since the applied AC penetrates the capacitive cell membranes (CM), delaying the applied current [9, 10].

## Phase-sensitive bioimpedance instruments and low intrinsic impedance surface electrodes: mandatory for L-BIA measurement

There is no international consensus for the electronic design of instrumental systems for non-invasive or minimally invasive BI measurements for use in *in vivo*, *ex vivo*, and *in vitro* studies [6, 11–13]. There is also no consensus for estimation of body composition in healthy paediatric populations [8, 14]. The lack of international consensus results in problems: 1) the intrinsic impedance of the skin contact electrode is not specified, nor are the characteristics of the electrode, which are required for compatibility with technical design aspects of the BI device; 2) it is not possible to directly obtain the measurement of R and Xc due to a lack of disclosure of the error in the measurement of R and Xc; and 3) incomparability of BI measurements from devices that measure individuals in standing the traditional supine decubitus position, thus ignoring the effects of gravity of fluid distribution. These factors contribute to a lack of interchangeability of BI values among instruments made by different manufacturers.

Technical factors and body position differentiate BIA measurements among devices and test protocols. Bogónez-Franco et al. [13] performed whole-body (wrist to ankle), segmental and localized measurements using BioparHom Z-Métrix (BioparHom, Bourguet du lac, France) and Impedimed SFB7 (Impedimed, Brisbane, Australia) from 4 kHz to 1 MHz. The error between devices was greater for Xc than R at lower compared to a higher frequency. At 50 kHz, the maximum difference between Z-Métrix and SFB7 in Xc was ~12% for whole-body, ~35% for segmental and ~14% for L-BIA.

Other studies have been less specific and have not included inter-device variability in Xc measurements. Ward [11] compared Xitron 4200, Impedimed SFB7 and SEAC SFB3 for the assessment of body composition using 10 to 500 kHz and the ratio of low to high frequency R values. Chumlea et al. [15] compared whole-body (wrist to ankle) 50 kHz BI measurements obtained with a Valhalla 1990B (San Diego, CA) and a RJL BIA-101 (Detroit, MI) phase-sensitive device and found an overestimate of R of approximately 10 Ω with the Valhalla device. Thus, differences in BI measurements prohibit interchangeability of measurement values between BI devices.

When using a phase-sensitive BI device, the intrinsic impedance of cutaneous surface electrodes is responsible for inducing errors in the phase detection, hence Xc values [6, 12, 13]. The consequence of a high intrinsic impedance is misinformation on hydration, nutritional status, or tissue integrity. Avoidance of the confounding effect of skin-to-electrode contact associated with high intrinsic impedance contact electrodes with needle electrodes may explain the success of electrical impedance myography in the identification of muscle pathology in patients with neuromuscular diseases [16, 17]. However, the practicality of using needle electrodes is problematic and limits general use of this method.

The lack of consensus on the appropriate level of intrinsic impedance for Ag/AgCl electrodes limits the ability to obtain valid BI measurements. Shiwei et al. [18] found wide variability in BI measurements, using 5 commercial Ag/AgCl electrodes on healthy volunteers. Nescolarde et al. [19] reported large variability of intrinsic R (11 to 665 Ω) and intrinsic Xc (0.25 to 2.50 Ω) of commercial Ag/AgCl electrodes. Use of surface electrodes with variable intrinsic impedances and the same 50 kHz phase-sensitive BI instrument adversely influences the classification of hydration in healthy adults [19].

An important moderating factor is the phase-sensitivity of a BI instrument. Genton et al. [20] surveyed PhA measurements of elderly healthy adults, hospitalized and ambulatory patients measured with a variety of 50 kHz BI instruments. They reported an underestimation of nearly 1° in PhA measurements with non-phase-sensitive BI devices.

Another concern is body position during the BI measurement. An incomparability is evident in BI measurements from devices that use the traditional supine decubitus position compared to standing upright, thus ignoring the effects of gravity of fluid distribution. The principal effect is a significant decrease of 1° in PhA measurements in the standing compared to the supine position [21, 22].

Each of these factors, individually or in combination, in a BI measurement system influences the validity, sensitivity and specificity of clinical applications for an individual patient. Thus, for optimal measurement of passive BI characteristics and physiological interpretations, standardization of the BI instrument and electrodes is required.

## Translating tetrapolar 50 kHz bioimpedance measurements into physiological equivalents: evaluation of different validation models of cell membrane damage

Lukaski [23] provided the first experimental evidence relating bioimpedance measurements to biological variables using the cell model in Fig. 1B, C. The simple experiments used a 50 kHz phase-sensitive bioimpedance instrument with four electrode systems and reported R and Xc in russet potatoes before and after microwave cooking with different testing models. Raw and cooked whole potatoes first were measured using needle electrodes then homogenous samples of the potatoes were measured in a simple conductivity cell to minimize the error in measurement due to the size and shape of the whole potatoes. Results were consistent and independent of the testing model. Cooking decreased Xc 100% regardless of the testing model, and R decreased more in the whole potato compared to the conductivity cell (76 and 54%). These findings led to the conclusion that the reduction of Xc to 0 indicated a disappearance of capacitance due to the disruption of cell membranes and the substantial reduction in R may be attributed to an expansion of ECW subsequent to the disrupted cell membranes. These observations provide a basis for the investigation of PhA in body composition (e.g., lean soft tissue mass and BCM), prognosis, muscle injury, and sarcopenia.

**Fig. 3:**
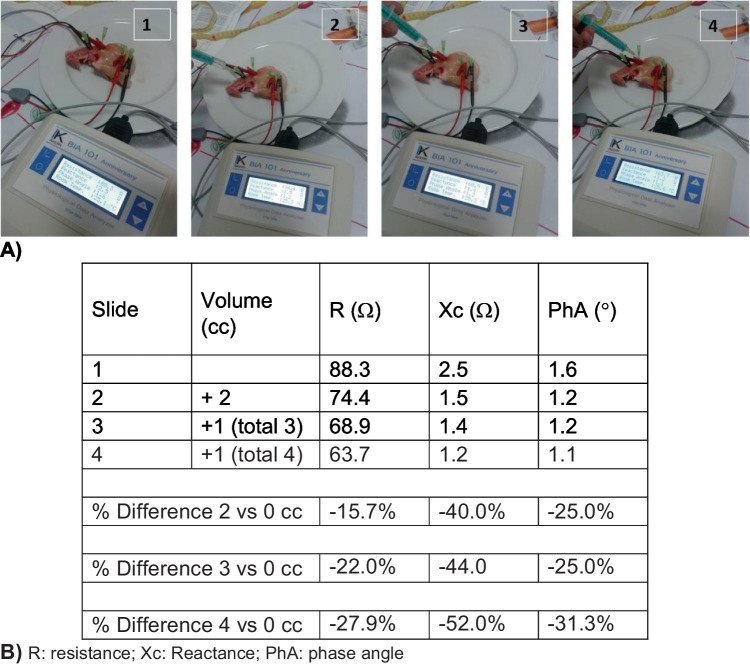
Tetrapolar bioimpedance measurement on a leg of poultry using needle electrodes (**A**). The R, Xc, and PhA reference values (1), after 2 cc of saline solution (2), after 3 cc (3), and after 4 cc of saline solution (4) (**B**)

Other experiments contributed to the hypothesis that L-BIA determinations of R, Xc, and, PhA could be predictors of disruptions in localized fluid distribution and cell membrane integrity. One experimental model used consecutive infusions of saline (2, 3, and 4 cc increments) into the lower limb of poultry with paired serial tetrapolar phase-sensitive bioimpedance with four needle electrodes (Fig. 3A). Progressive accumulation of exogenous saline resulted in a sequential decrease in R (Fig. 3B), which is consistent with increased conductivity in the extracellular space (e.g., R is inversely related to conductivity). The changes in Xc may be the result of plasma entering the muscle (meat) cells that produces a vesicle (intra- or intermuscular gap) filled conductive fluid and consequently a gradual decrease in Xc (40 to 42%). This gap-filled alteration in muscle structure, however, was more evident when the volume of fluid reached 4 cc (52%). Interestingly, PhA decreases were stable (25%) until 4 ccs of saline were injected (> 31%). These data emphasize that localized fluid accumulation disproportionately affects R and Xc with lesser effects on PhA.

**Fig. 4:**
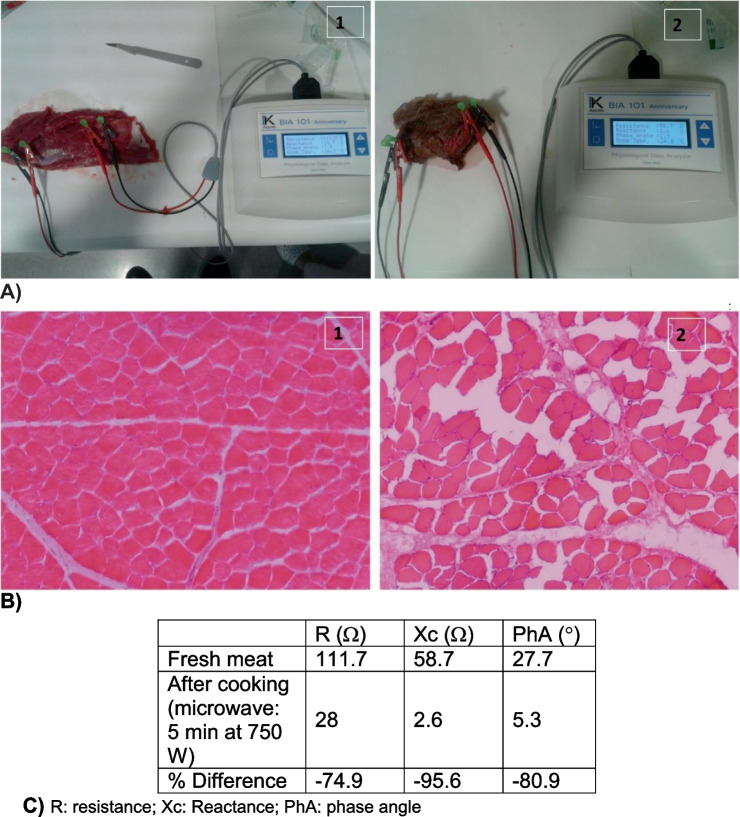
Tetrapolar phase-sensitive bioimpedance measurement using needle electrodes obtained from *vastus lateralis* skeletal muscle of an adult beef after cooking (**A1**) and before cooking using microwave 5 min at 750 W (**A2**). Histological images of fresh meat after cooking (**B1**) and before cooking using microwave 5 min at 750 W (**B2**). The R, Xc, PhA before and after cooking and % of the difference with respect to fresh meat after cooking (**C**)

An additional experiment (Fig. 4) was carried out on a piece of fresh meat obtained the from *vastus lateralis* skeletal muscle of an adult bovine before and after cooking using a microwave oven applying 750W for 5 min (Fig. 4A). Histological analysis of the meat before and after cooking (Fig. 4B) confirms the results obtained using tetra-polar L-BIA measurements (Fig. 4C) revealing damage to connective tissue with disruption of the cellular membrane and extracellular matrix (gap-filled).

Another experiment using a meat specimen provided additional support to the hypothesis that L-BIA R is related to localized fluid changes (extracellular) and Xc to localized gap-filled muscle injury (cellular membrane disruption). The protocol included a piece of beef obtained immediately after slaughter with physiological serum injected into the fresh meat to simulate the edema that appears in a gap-filled muscle injury. Standardized tetra-polar L-BIA measurements and histological examinations were carried out in the laboratory to identify structural changes after cooking.

The three basic experiments in different biological samples support the hypothesis that at 50 kHz, R is related fundamentally with extracellular fluid and Xc to the integrity of cell membrane (gap-filled), this last is universally associated with the CM.

## Localized bioimpedance in wound healing

The first clinical application of L-BIA was the non-invasive monitoring of wound healing. Studies of cells in culture found that R increased significantly during the development of a functional monolayer, decreased significantly with disruption of the monolayer, and serially increased with the progressive restoration of the monolayer [24]. Tetrapolar phase-sensitive 50 kHz impedance measurements at the trochanter and coccyx of adults at different levels of risk for pressure wounds were evaluated to categorize the importance of L-BIA measurements to estimate the risk of pressure ulcers in hospitalized adults [25]. Patients at high risk for pressure ulcers, compared to other patients, not at risk, had significantly reduced Xc, R, and PhA values that were indicative of risk of malnutrition, extracellular water (ECW) accumulation, and reduced cell membrane integrity. Multiple linear regression analysis identified Xc and serum albumin as significant predictors of risk for pressure ulcers (R^2^ = 0.96).

Serial L-BIA measurements of lower limb wounds described the first evidence of changes in R, Xc, and PhA associated with normal healing and complications [25]. Contact adhesive low intrinsic impedance Ag/AgCl electrodes were positioned in standardized locations relative to each open wound of varying length, width, and depth. Uncomplicated wound healing exhibited a common pattern of serial bioimpedance measurements. After initial treatment, R increased for a variable duration specific to the severity of the wound, decreased transiently due to erythema and then increased progressively with further epithelialization. Reactance increased briefly then stabilized as cell layers become contiguous. Phase angle exhibited a pattern similar to that observed with Xc. The general pattern was longitudinal increases in R, Xc, and PA (11, 90, 50%, respectively) during uncomplicated healing that is consistent with decreased ECW, increased cell mass and vitality, and epithelialization.

Medical treatments and procedures administered to facilitate wound healing directly and acutely affected impedance parameters [26]. Debridement of a nonhealing wound produced a transient decrease in R, Xc and PhA (2, 28, and 30%, respectively) that indicated a distinct disruption of tissues and cell membranes with a temporary increase in ECW. Trauma to tissues associated with wound preparation and application of the skin graft resulted in decreased R, Xc, and PA values (23, 65, and 59%, respectively) indicating a greater disruption of tissue and cell architecture and ECW expansion. Marked increases in R, Xc, and PA values (28, 210, and 178%, respectively) after recovery from the graft procedure suggested a substantial increase in the number of cells, cellular adhesion and wound repair.

Infection of wounds elicited distinct changes in the magnitude and direction of L-BIA measurements [26]. In contrast to debridement, methicillin-resistant Staphylococcus aureus infection of a wound produced a substantial decrease in R, Xc, and PhA (16, 63, and 56%, respectively) that altered the anticipated temporal increase in the L-BIA measurements during healing. Importantly, the direction and rate of change in R, Xc, and PhA signalled the presence of infection before detection with laboratory methods. This pattern suggests that infection increases ECW and decreases cell numbers and vitality.

A significant advantage of the use of *in vivo* L-BIA measurements for monitoring wound healing is the low within-patient variability (< 2 Ω for R and Xc; < 0.2° for PhA), and high test–retest reliability (r = 0.99). These low estimates of variability, and hence very high precision of measurement, ensure the L-BIA is capable to identify the physiological perturbations (20 to 200%) in L-BIA measurements associated with the pathology of soft tissue injury and potential complications with treatment and infection.

## Tetrapolar non-invasive 50 kHz phase-sensitive BI measurements including PhA assessment of muscle integrity and fluid localization in soft tissue injury

**Fig. 5:**
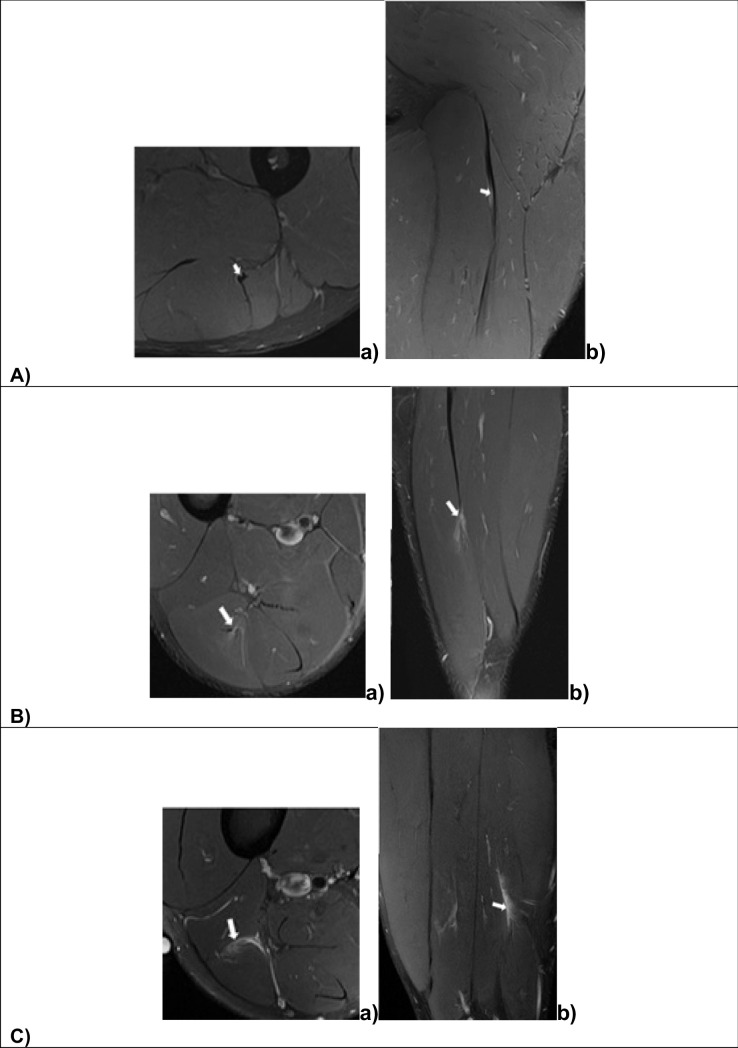
Magnetic resonance images of traumatic muscle injuries according to anatomical location: Tendinous injury of the central tendon of the hamstrings (arrow). There is a longitudinal tear of the central tendon with slight peritendinous oedema in axial (**a**) and coronal (**b**) intermediate weighted fat-suppressed images (**A**), proximal myotendinous junction (MTJ) injury grade 1b of long head of biceps femoris in axial (**a**) and coronal (**b**) intermediate weighted fat-suppressed image. Tear of the intramuscular aponeurosis (arrow), resulting in mild loss of tension and feathery oedema (**B**), and distal myofascial junction (MFJ) injury of long head of biceps femoris (arrow) in axial (**a**) and sagittal (**b**) intermediate weighted fat-suppressed image. There is epimysia tear, extensive feathery and intermuscular oedema (**C**)

Following the initial description of L-BIA to monitor uncomplicated wound healing and complications [26, 27] proposed the classification of skeletal muscle injury and validation with ultrasound (US) and magnetic resonance imaging (MRI). Structural damage in traumatic muscle injury (Fig. 5) can occur in diverse anatomical locations [intra-tendinous, myotendinous junction (MTJ), or myofascial junction (MFJ)] that require sophisticated radiological technology and analytic methods for identification and classification [e.g., The British Athletics Muscle Injury Classification (BAMIC)] [28]. The innovative L-BIA method offered a practical approach to facilitate the identification and grading of traumatic muscle injury.

### L-BIA method: electrode placement and controls for muscle injury studies

**Fig. 6:**
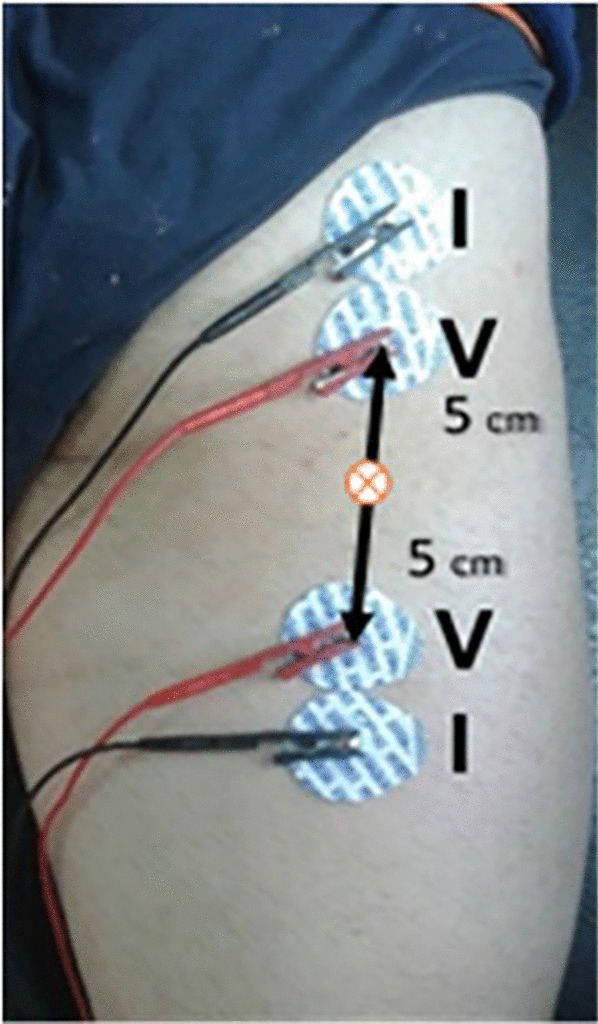
Electrode placement for short L-BIA configuration (I: pairs of current injection electrodes, V: pairs of detecting voltage electrodes)

Electrode quality and placement are critical to achieving reliable L-BIA measurements. Two pairs of contact adhesive Ag/AgCl electrodes with an intrinsic R of 11 Ω and intrinsic Xc of 0.30 Ω, (COVIDIEN Ref. 31050522, COVIDIEN LLC, Mansfield, IL, USA) are positioned as shown in Fig. 6 taking into account L-BIA short configuration. The L-BIA configurations could be short (muscle injury is anatomically located far from the bone) or large (muscle injury is anatomically located near the bone) [27, 29]. In a short L-BIA configuration the voltage detector electrodes (V) are placed 5 cm proximally and 5 cm distally, respectively, from the center of the injury while in a large L-BIA configuration, voltage electrodes are placed 10 cm proximally and 10 cm distally, respectively, from the center of the injury. In muscle injuries evaluated using L-BIA, the center of the injury is located by US. In both, short and large configurations, two pairs of current injection (I) electrodes are located close to the voltage electrode (Fig. 6). This placement is valid for circular snap electrodes, in the case of rectangular electrodes [19] is mandatory 2 cm between the current injecting electrode (I, black) and the voltage drop detecting electrode (V, red). The measurements were taken with a phase-sensitive impedance instrument (BIA 101 Anniversary, Akern-Srl, Florence, Italy) applying a constant sinusoidal alternating current of 245 µA RMS at 50 kHz. Validation of BI measurements of this instrument are determined using a parallel circuit made out with a precision reference resistor and capacitor, resulting in technical error measurement (variability) less than 1 Ω for R and < 2% for CAP.

To avoid errors in the repeatability of the L-BIA measurements, the first measurement is obtained 24-h after the injury to allow for some stabilization in the acute response to injury. For consecutive L-BIA measurements to track recovery and response to treatment specifically for an assessment of the time of return to play (RTP), it is mandatory to mark (draw) the area of electrode placement with a reliable permanent marker. These serial measurements require the use of a phase-sensitive 50 kHz BI analyzer and low intrinsic impedance electrodes [19].

A fundamental concern in application of L-BIA in muscle injury research is identification of muscle groups at high risk of injury. Among professional athletes, particularly football players, lower body muscle distribution tends to be symmetric between legs to prevent injury; soft tissue injury directly affects muscle symmetry. Therefore, to assess muscle injury and monitor effects of treatment of leg muscle injuries, it is imperative to use the contralateral, non-injured leg as a reference (control) for L-BIA measurements [29].

The protocol for the L-BIA testing procedure is the following:

1. Perform L-BIA measurements 24-h after injury (previously diagnosed by MRI) on the injury side and on the contralateral non-injury side of interest.
  This first and only measure on the contralateral non-injured side is taken as a reference during subsequent L-BIA measurements until RTP.
  To avoid errors in the placement of the electrodes during subsequent L-BIA measurements is mandatory to shade with a permanent marker the edge of the electrodes.
2. Repeat the L-BIA measure until the RTP on the injury side only.
  This must be so because, during the injury recovery time, the player will make biomechanical compensations that will affect the initial reference measurement of the non-injured contralateral side.
3. Determine the percent difference [% difference = 100% X (injured – non-injured)/non-injured] between the injured side with respect to the non-injured side (reference) of the successive L-BIA measurements.
  The % difference value represents the degree or magnitude of the injury's impact on the muscle and tissue structure and fluid distribution bioelectric characteristics.

Following a correct methodology in the placement of the electrodes in repeated L-BIA measurements to ascertain the return to play (RTP), it is crucial to compare the results of L-BIA with the progress of the injury healing using reference to the MRI and/or the US images as well as the self-reported symptoms by the injured athlete.

### L-BIA value of muscle injury according to muscle gap and return to play classification of football players

Sport-related muscle injury classifications rely on radiological imaging criteria from US and MRI. However, the prognosis using these criteria is unclear. Empirical evidence demonstrates that RTP depends on the severity of an injury [30], which is related to muscle gap defined as the retraction of muscle fibers shown in MRI images. An associated factor is the amount of edema in the injured muscle which is associated with a longer recovery time but the reliability is uncertain with MRI data [31, 32]. Valle et al. [33] proposed a new injury classification system based on muscle gap, which is limited by a lack of general availability of MRI. The L-BIA can overcome this practical limitation.

A pilot study of injured professional footballers revealed lower limbs injuries previously diagnosed by MRI 24-h after grades I, II, and III injury [27]. Compared to the non-injured leg, 24-h post-injury R, Xc, and PhA values decreased with increasing muscle severity injury [grade III (23, 45 and 28%), grade II (21, 32 and 12%) and grade I (12, 24, 12%). These findings suggest that decreases in R reflect localized fluid accumulation, and reductions in Xc and PhA indicate disruptions in cell membranes resulting in muscle cell and tissue injury. Successive post-injury L-BIA measurements revealed the time to achieve non-injury R, Xc, and PhA values with MRI confirmation of healing and RTP was inversely related to injury severity (grade III, 75 d; grade II, 30 d and grade I, 9 d). A second study confirmed and extended these preliminary observations [29]. Consistent with epidemiology of muscle injuries in football, injuries occurred in quadriceps, hamstrings, adductor and calf muscles, ranging in severity grade I, II and III previously diagnosed by MRI 24-h after injury. Regardless of MRI-defined injury severity, muscle injury predominantly effected Xc (grade I, 18; grade II, 33, grade III, 53), which directly resulted in reductions in PhA (grade I, 9; grade II, 17, grade III, 43). This substantial effect on Xc represents a pattern of soft tissue injury related to severity of muscle injury. The decreases in R, which indicate fluid distribution, were not proportional to severity of muscle injury (grade I, 10%; grade II, 18%; grade III, 14%). Disruption of muscle structure, evidenced by L-BIA measurements, increased with severity of muscle injury.

Nescolarde et al. [34] determined the relationship between muscle injury severity based on muscle gap and RTP in professional football athletes. Degree of oedema and gap presence were the criteria for injury severity of the muscle gap injuries: grade I, small area of edema [5–10% muscle injury cross-sectional area (CSA)], < 5 cm length and no gap; grade IIf, moderate area of oedema (10–50% CSA, 5–15 cm length, feathered image and no gap; grade IIg, moderate area of oedema (10–50% CSA, 5–15 cm length, feathered image and a gap. Increased oedema in muscle was seen with significantly greater % differences in R (10, 13 and 20%). The combination of increased oedema with presence of muscle gap produced significantly greater % differences in Xc (13, 24, 48%) with similar but smaller deficits in PhA (3, 11, 21%). Thus, the most significant changes were observed in the severe injuries (oedema and muscle retraction) with the highest statistical significance in Xc and PhA. The functional consequence of the effects on Xc and PhA was a significantly (p < 0.001) delayed RTP among the athletes with the greatest injury severity derived from L-BIA measurements (8, 14, and, 48 d). These findings reveal that the grade of muscle injury is proportional to the size of the muscle gap, and the gap in association with localized fluid accumulation directly affects the restoration of muscle function based on RTP.

Table 1 summarizes the effect of injury severity and presence of muscle gap, defined as the relative decreases in Xc, R, and PhA of the injured side value compared to the non-injured leg, of L-BIA measurement values [34]. Increasing grade of muscle injury and presence of muscle resulted in the greatest disparity (decrease) in L-BIA predominantly in Xc and PhA.

**Table 1:** Localized bioimpedance determinations of percent (%) difference (decrease) between injured side respect to non-injury side according to the muscle gap and return to play (RTP)

Classification of Muscle Injury according to Muscle Gap	% Difference Muscle gap			RTP, d
	R	Xc	PA	
Grade 1 A small area of edema, either myotendinous or myofascial with no gap	~10	~13	~3	~8
Grade 2f A moderate amount of edema with feather-like image and no gap	~13	~24	~11	~14
Grade 2 g A moderate amount of edema with feather-like and gap image	~20	~48	~21	~48

Grade 1: < 10% of cross-sectional area (CSA) of the muscle affected and < 5 cm of craniocaudally length with no gap

Grade 2f: 10–50% of CSA and 5–15 cm of length with feather-like image and no gap

Grade 2 g: 10–50% of CSA and 5–15 cm of length with feather-like and gap image

Adapted from Nescolarde et al. [34]

Nescolarde et al. [35] discriminated differences in L-BIA measurements between myotendinous junction (MTJ), and myofascial junction (MFJ) that are usually injury in professional football players. Intra-tendinous injuries are purely tendinous injuries and do not affect the myo-connective junction (Fig. 6A). Myotendinous junction (MTJ) injury includes the myo-connective junction, specifically at an aponeurosis or a tendinous expansion attached to muscle fibers (Fig. 6B). Myofascial junction injury (MFJ) involves muscle fiber antithesis perimysium and/or epimysium (Fig. 6C).

Table 2 summarizes the L-BIA measurements of complex muscle–tendon injuries [29, 36]. The most important finding was a significant decrease (P ˂ 0.01) of the L-BIA parameters R, Xc, and PA, in the inter MTJ and MFJ, and intra MTJ and MFJ injuries (Table 2) [35]. Similar results were found when MTJ were classified in the different grades of MTJ injuries. Importantly, Xc predominantly (-12 to -34% difference) and, to a lesser degree, PhA (-4 to -17% difference) differentiated severity of the MTJ injuries. However, reliable detection of intra-tendinous injuries is hampered (Table 2) by the complex composition of the tendon [compact structure of the tendon with tenocytes attached to a highly ordered fibrillar collagen matrix composed of type-I collagen (65–80% of its dry mass)] and small leucine-rich proteoglycans that regulate collagen self-assembly into collagen fibrils and contribute to collagen fibers [37]. This heterogeneous matrix offers a very high impedance and the changes produced by the muscle injury are impossible to detect through serial changes in R and Xc measurements.

**Table 2:** Localized bioimpedance characterization of muscle injury severity according to the anatomical location with time to return to play

Classification of muscle injury according to anatomical location	% Difference Muscle gap			RTP, d
	R	Xc	PhA	
Intra-tendinous	~2	~0.5	~1	~72
MTJ-Grade 1	~8	~12	~4	~8
MTJ-Grade 2	~9	~20	~11	~14
MTJ-Grade 3	~17	~33	~18	~52
MFJ	~20	~34	~17	~20

Adapted from Nescolarde et al. [35]

*MTJ* myotendinous junction, *MFJ* myofascial junction, *RTP* return to play

The degree of MTJ injury effected (p < 0.001) the RTP (Table 2). Grade 1 resulted in less time to RTP compared to Grade 3, which required more time than Grade 2. Assessments using L-BIA identified injuries in the myo-connective junction consistent with MRI imaging. This type of muscle injury (MTJ-Grade 3) has a poor prognosis with a longer time to RTP (52 d) and could present a greater risk of re-injury compared to other injuries [38, 39].

The L-BIA method enables the detection and grading of severity of MTJ and MFJ injuries. The sensitivity of L-BIA method is shown to be higher in the percentage change (% decrease) of Xc 24 h after injury, which is related to muscle cell disruption. In addition, L-BIA measurements 24 h after injury shows a concordance between grades 1, 2 and 3 of the MTJ injuries and days to RTP.

## Conclusion and future research

Localized BIA is a practical method to identify, grade severity, and monitor healing of injured muscle. Use of a 50 kHz phase-sensitive BI instrument with low intrinsic impedance contact electrodes results in low technical error of measurement that permits reliable detection of significant decreases in series-equivalent R, Xc, and PhA values of an injured ROI compared to an uninjured contralateral site. The principal effects of muscle injury are seen with Xc and PhA as % differences (relative decreases) that are inversely related to the radiologically-determined severity of injuries and the practical outcome of return to training and competition for athletes. Empirical evidence from experiments involving cooking of vegetable and meat specimens demonstrates major reductions in Xc and PhA that correspond to histological evidence of cell membrane destruction. Graded infusion of saline into meat specimens reveals large increases in the extracellular space with decreases in R and Xc comparable to plasma entering the muscle (meat) cells that associated with an intra- or intermuscular gap filled conductive fluid. Decreases in PhA were observed only with greatest fluid volume. These findings highlight that localized fluid accumulation disproportionately affects R and Xc with lesser effects on PhA.

Whereas all commercial BI instruments provide series measurements, a more physiological representation of cells and fluids is the parallel-equivalent model. This model utilizes basic mathematical transformation of series BI measurements into parallel values. It emphasizes parallel Xc, which is frequency-dependent and inversely related to membrane CAP. Capacitance is a measure of the storage of applied AC by cell membranes and is interpreted as an index of cellular membrane health in whole-body and body composition applications. Thus, the substantial decreases in Xc identified with graded muscle injury indicate adverse changes in cell membrane structure. Important research findings reveal important physiological associations with CAP. *In vitro* studies of varying concentrations of red blood cells in a constant volume of saline demonstrate that CAP is directly related to HCT. Importantly, series Xc, indicates “cells in a watery” environment. This inter

pretation suggests an important insight in the assessment of hydration status *in vivo* using whole-body BI measurements. Another extension of this research is the relationship between CAP and ^40^ K whole body counting and resting metabolic rate (RMR). Strong statistical associations suggest that CAP is related to body cell mass (BCM).

Opportunities for future research based on these results should include monitoring of muscle structure after surgery and during rehabilitation, establishing normative ranges of PhA for identification of risk of sarcopenia, and tracking structural changes in muscle during weight loss and progress with interventions to ameliorate risk of malnutrition. Additional research is needed to determine the validity of series Xc to monitor hydration in individuals at risk for hypohydration including physically active individuals and the elderly.

## Abbreviations

AC: Alternating current
tan^−^^1^: Arc tangent
BI: Bioelectrical impedance
BCM: Body cell mass
CAP: Capacitance
cm: Centimeter
cc: Cubic centimeter
d: Day
°: Degree
ECW: Extracellular water
HCT: Hematocrit
h: Hour
Z: Impedance
ICW: Intracellular water
kHz: Kilohertz
L-BIA: Localized bioimpedance
μA: Microamps
min: Minute
MF: Mutiple frequency
MFJ: Myofascial junction
MTJ: Myotendinous junction
Ω: Ohm
PhA and Φ: Phase angle
Xcp: Parallel reactance
Rp: Parallel resistance
^40^K: 40-Potassium
ROI: Region of interest
RC: Resistor-capacitor model
RMS: Root mean square
Xc: Series reactance
R: Series resistance
Ag/AgCl: Silver/silver chloride
SF: Single frequency
TBW: Total body water
W: Watt
